# Editorial: Methods and applications in frontiers in neuroanatomy

**DOI:** 10.3389/fnana.2023.1256867

**Published:** 2023-08-29

**Authors:** Clément Ricard, Lidia Alonso-Nanclares, Basilis Zikopoulos, Martin Oheim

**Affiliations:** ^1^Université Paris Cité, CNRS, Saints-Pères Paris Institute for the Neurosciences, Paris, France; ^2^Laboratorio Cajal de Cirtuitos Corticales, Instituto Cajal, CSIC, Madrid, Spain; ^3^Centro de Tecnología Biomédica, Universidad Politécnica de Madrid, Madrid, Spain; ^4^Human Systems Neuroscience Lab, Department of Health Sciences, College of Health and Rehabilitation Sciences: Sargent College, Boston University, Boston, MA, United States; ^5^Department of Anatomy and Neurobiology, Boston University School of Medicine, Boston, MA, United States

**Keywords:** neuroanatomy and morphometry, human brain, stereotaxic, immunochemistry, post-mortem

## Introduction

Over the course of History, neuroanatomical knowledge has evolved in many ways from philosophical conceptions, over artistic illustrations to medical applications. From human dissections initiated by Andreas Vesalius to modern non-invasive 3-D neuroimaging techniques, our knowledge of the inner structure and function of the nervous system has relied on the continuous development of new methods. In this Research Topic, we have gathered some of the latest innovations that continue to push the frontiers of the neuroanatomy. For this theme issue “*Methods and applications in frontiers in neuroanatomy*”, we have selected papers that range from (1) novel approaches to collect, fix, handle and analyze human brains; (2) histological protocols and their applications and (3) approaches to study specific tracks in the nervous system.

## Novel approaches to collect, fix, handle, and analyze human brains

In their study, Insausti et al. propose a new protocol for *in-situ* fixation of the human brain compatible with a broad range of applications, including fluorescence and electron microscopy, *ex-vivo* MRI and classical anatomical studies. Their protocol optimizes human brain tissue fixation, facilitates imaging lacking distortion, and allows detailed analysis both in control and pathological conditions.

García-Cabezas et al. describe a new method for the stereotaxic cutting of post-mortem human brains for anatomical studies. The rationale behind their work is that, in general, the brains obtained from patients are not presented in stereotaxic coordinates. They developed a newly crafted instrument that can be built in any laboratory and that enables the registration of human brain samples in the stereotaxic space of Talairach and Tournoux, which in our belief is an important step forward for reproducibly navigating and finding structures with precision.

## Histological protocols and their applications

The article of Muniz Partida and Walters presents a free-float immunohistochemistry protocol with paraffin embedded tissue that saves time, resources and tissue. Using mouse brain, they also demonstrate that their method is compatible with both chromogenic and fluorescence detection methods.

From an applicative point of view, Elhessy et al. show that conventional histological and chromogenic immunohistochemical approaches are reliable tools for the evaluation of pharmacological treatments. In their article, they evaluate the neuroprotective effects of three substances on a rat model of sciatic nerve injury.

Using conventional histological stainings in macaque cortex, computational analysis, and MRI scans John et al. present a systematic variation between weakly and sharply laminated cortices that they describe as the cortical spectrum.

## Approaches to study specific tracks in the nervous system

Neuroanatomical methods do not only rely on protocols and experiments done on the bench. Gallet et al. show that conventional systematic reviews can help to gather disseminated information to highlight the role of specific structures in the brain. They describe the Frontal Aslant Tract, an anatomically symmetric white-matter tract with functional asymmetry that should be considered during surgery and perioperative monitoring.

Finally, Shim et al. address the limitations associated with quantifying myelin content using MRI using a single ME-MP2RAGE sequence.

## Conclusions

The field of neuroanatomy continues evolving and growing through the development of new methods and approaches, based on new ideas and discoveries that arise both from novel technologies as well as from the adaptation and improvement of established protocols. This Research Topic reflects this duality and the fact that frontiers can always be expanded.

## Author contributions

CR: Funding acquisition, Validation, Writing—original draft, Writing—review and editing. LA-N: Validation, Writing—original draft, Writing—review and editing. BZ: Validation, Writing—original draft, Writing—review and editing. MO: Funding acquisition, Validation, Writing—original draft, Writing—review and editing.

